# Re: Nielsen N.K, Hjort P.B, Hansen E.B, et al. Substaging and stratified treatment of T1 bladder cancer – a nationwide register‑based study. Eur Urol Open Sci 2026;90:92‑100

**DOI:** 10.1016/j.euros.2026.08.008

**Published:** 2026-09-17

**Authors:** Xin Li, Yapeng Xing

**Affiliations:** a Department of Urology, The Third Affiliated Hospital of Kunming Medical University, Yunnan Cancer Hospital, Peking University Cancer Hospital Yunnan, Kunming, China

We read with interest the nationwide study by Nielsen et al [1] evaluating Denmark’s pT1a/pT1b-guided management strategy for T1 bladder cancer. Among 3086 patients, pT1b disease was associated with poorer overall survival and a higher observed risk of progression than pT1a disease. Furthermore, 20% of the patients with pT1b who underwent early cystectomy had pT2 disease in the cystectomy specimen. These findings establish clinically relevant risk enrichment, but they do not establish pT1b as a predictive marker of benefit from immediate radical cystectomy.

This distinction is crucial because treatment was assigned partly according to the marker being evaluated, creating confounding by indication. Moreover, early cystectomy was performed in 41% of the patients with pT1b, all of whom were excluded from the recurrence and progression analyses, introducing selection bias. Residual confounding is also likely because tumor grade, size, multiplicity, concomitant carcinoma in situ, lymphovascular invasion, variant histology, and repeat resection findings were unavailable. These factors underpin contemporary risk-directed management [2].

The randomized Japan Clinical Oncology Group 1019 trial further illustrates the importance of repeat resection after an initial diagnosis of high-grade T1 disease [3]. If repeat resection can restratify risk, pT1b’s apparent prognostic signal may partly reflect undetected residual disease rather than substage per se. Because repeat resection findings were unavailable in the Danish registry, the independent actionable value of pT1b remains uncertain.

Emerging computational histology artificial intelligence assays, including those developed by Valar Labs, have been externally validated to stratify risks of recurrence, progression, and Bacillus Calmette-Guérin (BCG)–unresponsive disease after intravesical BCG treatment in high-risk non–muscle-invasive bladder cancer [4]. They may complement pT1 substaging and conventional clinicopathological assessment, but their role in guiding immediate cystectomy remains to be established.

For pT1b to be treatment predictive, it must modify the absolute benefit of immediate cystectomy rather than merely correlate with adverse outcomes. Demonstrating this would require treatment-stratified risks among clinically comparable patients and a prespecified substage-by-treatment interaction.

Two complementary analyses could address this question. First, the incremental value of pT1b beyond an established multivariable risk model should be evaluated using calibration and decision-curve analysis. Second, comparative effectiveness should be assessed through an explicitly specified target trial emulation [5] comparing immediate cystectomy with repeat resection followed by BCG or surveillance and response-triggered rescue cystectomy, with aligned eligibility, treatment assignment, follow-up, and clinically relevant outcomes.

The 20% upstaging rate makes pT1b an important diagnostic alarm. However, without evidence of treatment–effect heterogeneity or net clinical benefit, it should not be interpreted as proof that pT1b alone identifies patients who benefit from immediate cystectomy.

  ***Conflicts of interest:*** The authors have nothing to disclose.
